# A Small Regulatory RNA Controls Cell Wall Biosynthesis and Antibiotic Resistance

**DOI:** 10.1128/mBio.02100-18

**Published:** 2018-11-13

**Authors:** Jessica Borgmann, Sina Schäkermann, Julia Elisabeth Bandow, Franz Narberhaus

**Affiliations:** a Department of Microbial Biology, Ruhr University Bochum, Bochum, Germany; b Department of Applied Microbiology, Ruhr University Bochum, Bochum, Germany; University of Würzburg

**Keywords:** antibiotic resistance, gene regulation, plant-microbe interaction, posttranscriptional control, regulatory RNA

## Abstract

Small regulatory RNAs play an important role in the adaptation to changing conditions. Here, we describe a differentially expressed small regulatory RNA (sRNA) that affects various cellular processes in the plant pathogen Agrobacterium tumefaciens. Using a combination of bioinformatic predictions and comparative proteomics, we identified nine targets, most of which are positively regulated by the sRNA. According to these targets, we named the sRNA PmaR for peptidoglycan biosynthesis, motility, and ampicillin resistance regulator. Agrobacterium spp. are long known to be naturally resistant to high ampicillin concentrations, and we can now explain this phenotype by the positive PmaR-mediated regulation of the beta-lactamase gene *ampC*. Structure probing revealed a spoon-like structure of the sRNA, with a single-stranded loop that is engaged in target interaction *in vivo* and *in vitro*. Several riboregulators have been implicated in antibiotic resistance mechanisms, such as uptake and efflux transporters, but PmaR represents the first example of an sRNA that directly controls the expression of an antibiotic resistance gene.

## Abstract (precis)

The alphaproteobacterium Agrobacterium tumefaciens is able to infect various eudicots causing crown gall tumor formation. Based on its unique ability of interkingdom gene transfer, Agrobacterium serves as a crucial biotechnological tool for genetic manipulation of plant cells. The presence of hundreds of putative sRNAs in this organism suggests a considerable impact of riboregulation on A. tumefaciens physiology. Here, we characterized the biological function of the sRNA PmaR that controls various processes crucial for growth, motility, and virulence. Among the genes directly targeted by PmaR is *ampC* coding for a beta-lactamase that confers ampicillin resistance, suggesting that the sRNA is crucial for fitness in the competitive microbial composition of the rhizosphere.

## INTRODUCTION

Small regulatory RNAs (sRNAs) or noncoding RNAs (ncRNAs) are versatile regulators crucial for bacterial adaptation to changing environments (1, 2). These small RNA molecules range between 50 and 500 nucleotides (nt) in length and usually remain untranslated. Most sRNAs bind to target mRNAs modulating their stability and/or translation, albeit protein activity can also be controlled by sRNAs (3). Thus, sRNAs are involved in the differential regulation of numerous cellular pathways, including cell division (4), stress responses (5), quorum sensing (6), and virulence (7, 8). Moreover, several sRNAs have an impact on antibiotic resistance by affecting the expression of genes coding for uptake or efflux systems, peptidoglycan biosynthesis, or biofilm formation (9). Two different types of sRNAs can be distinguished according to their location on the genome. While *cis*-antisense RNAs (asRNAs) are encoded on the opposite strand of their target gene (10), *trans*-encoded sRNAs are located in intergenic regions and usually interact with several targets from distinct genomic locations (11). Since *trans*-encoded sRNAs share only limited base pair complementarity with their target mRNAs, their association is often promoted by RNA chaperones, such as Hfq (12).

Most of the previously described sRNAs have been studied in enterobacteria, such as Escherichia coli or Salmonella spp. (2, 3). However, by means of differential RNA sequencing, sRNAs have been identified in essentially all bacterial and archaeal species studied thus far (13, 14), including alphaproteobacteria, such as photosynthetic Rhodobacter species (15), plant-symbiotic rhizobia (16–18), and the mammalian pathogen Brucella abortus (19). The alphaproteobacterium Agrobacterium tumefaciens, also known as Agrobacterium fabrum (20), is a plant pathogen that has the unique ability to transfer part of its own DNA (T-DNA) into numerous eudicots (21). Integration of the T-DNA into the plant genome and subsequent expression of the involved genes leads to enhanced production of phytohormones and thereby to the formation of so-called crown gall tumors (22, 23). Through genetic engineering of Ti plasmids and their cognate T-DNA, A. tumefaciens has become the most important biotechnological agent for genetic manipulation of plant cells. As a member of the *Rhizobiaceae* family, A. tumefaciens is naturally resistant to certain β-lactam antibiotics, based on the chromosomally encoded beta-lactamase AmpC (24, 25). This enzyme is highly conserved among *Rhizobiaceae* and is possibly advantageous for microbial competition in the rhizosphere and the specific lifestyle of A. tumefaciens.

Recently, several RNA sequencing (RNA-seq) studies revealed more than 600 putative sRNAs in A. tumefaciens, suggesting a crucial role of sRNA-mediated regulation in this organism (26–29). At present, only a small number of Agrobacterium sRNAs have been functionally characterized. RepE was the first sRNA described in A. tumefaciens and controls Ti plasmid replication (30). The growth-phase-regulated sRNA AbcR1 targets multiple mRNAs of ABC transporter substrate-binding proteins, indicating an important role in nutrient acquisition during the transition to stationary phase (31). Importantly, AbcR1 was shown to regulate the uptake of γ-aminobutyric acid (GABA) (32), an amino acid derivative produced by wounded plants that stimulates degradation of a quorum sensing signal (33). The sRNA RNA1111 expressed from the Ti plasmid has an impact on the aggressiveness of the phytopathogen and affects the expression of several virulence genes (26).

To reveal the biological function of the countless bacterial sRNAs, one of the challenges in the field is the identification of their target genes due to the imperfect complementarity of the sRNA-mRNA pairs. Contemporary bioinformatic prediction programs can support target mRNA identification and currently work best for enterobacteria (34). The identification of sRNA targets in other species has remained difficult. Often, even experimentally verified sRNA-mRNA interactions are not predicted as top candidates by the available algorithms (35). In the present study, we used a combination of bioinformatic predictions and comparative proteomics by mass spectrometry to identify target mRNAs of the small regulatory RNA PmaR in A. tumefaciens. We identified PmaR as a crucial regulator for peptidoglycan biosynthesis, motility, and biotin synthesis. Moreover, PmaR regulates ampicillin resistance by modulating beta-lactamase levels. The major impact of PmaR on Agrobacterium physiology undermines the importance of sRNA-mediated regulation in this organism.

## RESULTS

### Expression of PmaR is induced in stationary phase.

**FIG 1:**
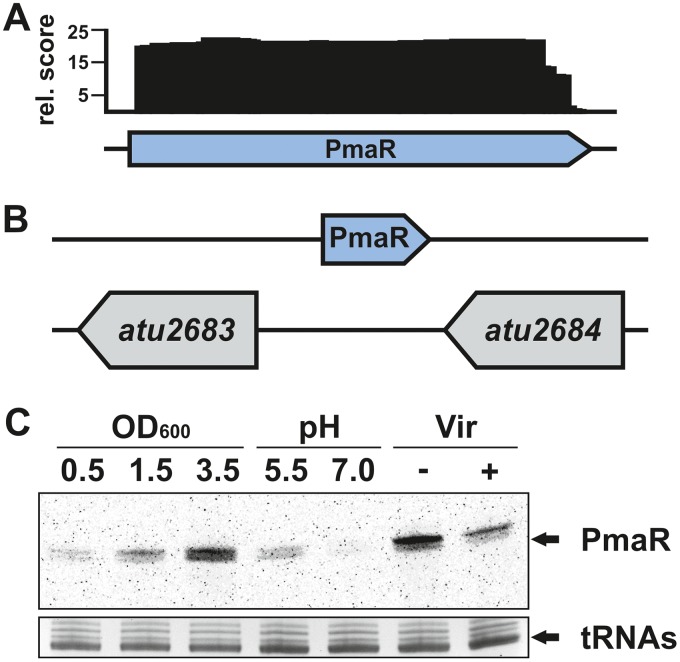
Expression and genomic context of PmaR. (A) Schematic drawing of mapped PmaR reads from dRNA-seq (−Vir libraries) (29). rel., relative. (B) Genomic location of PmaR on the circular chromosome. (C) Transcript amounts of PmaR detected by Northern blot analysis in *A. tumefaciens* C58. Samples were taken from cultures in YEB medium at different growth phases, in minimal medium at different pH values, and under nonvirulent (−Vir) and virulent (+Vir) conditions.

In previous work (29), PmaR (formerly C10) from the circular chromosome of A. tumefaciens was found to be transcribed under virulent and nonvirulent conditions (Fig. 1A). The gene of the *trans*-encoded sRNA is located between two hypothetical open reading frames of unknown function (Fig. 1B). Further RNA-seq data demonstrated that PmaR is highly expressed in complex medium at different growth phases (27). By Northern blot analysis, we observed differential expression of PmaR depending on growth conditions and growth phases (Fig. 1C). Transcript levels were highest during stationary phase in complex medium and in minimal medium (−Vir conditions). Furthermore, we observed higher expression of PmaR in minimal medium under acidic conditions (pH 5.5) than with neutral pH and downregulation of the sRNA under virulence-mimicking conditions. Differential expression of a sRNA often is indicative of a regulatory role, which motivated us to study the physiological role of PmaR.

### The structure of PmaR contains an accessible C-rich loop.

**FIG 2:**
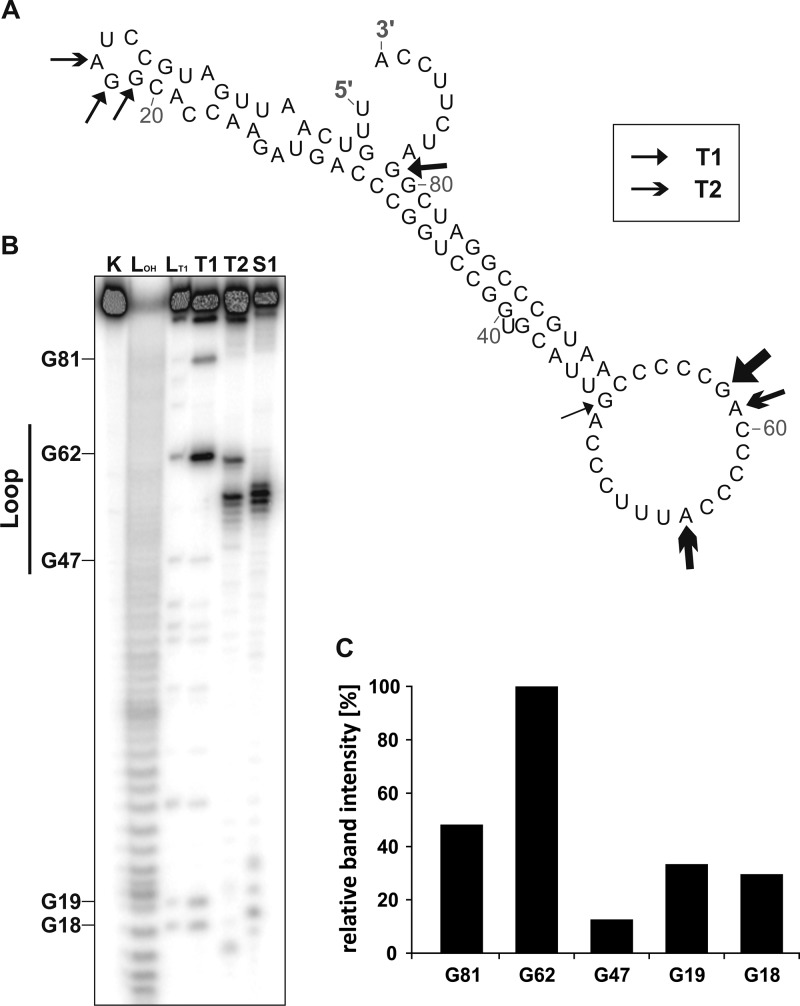
Structural features of PmaR. (A) Predicted secondary structure of PmaR by RNAfold (Institute for Theoretical Chemistry, University of Vienna [http://rna.tbi.univie.ac.at/]). Arrows indicate cleavage sites for RNases T1 and T2. (B) Enzymatic structure probing of PmaR with RNases T1 and T2 and nuclease S1. Lane K, unfolded RNA treated with RNase T1 served as a control. L_OH_, alkaline ladder; L_T1_, T1 ladder. (C) Quantification of cleavage products by RNase T1 derived from selected guanine residues by pixel counting using the AlphaEaseFC software (Alpha Innotec). The band intensity of the G62, the best substrate of RNase T1, was set to 100%.

The RNAfold-predicted secondary structure of PmaR comprises a spoon-like structure with a long stem and a single-stranded loop containing several C-rich regions (Fig. 2A). Enzymatic structure probing with RNases T1 (cuts single-stranded guanines) and T2 (cuts preferentially single-stranded adenines) and nuclease S1 (cuts the 3ʹ end of unpaired nucleotides) confirmed a highly stable structure, which was preferentially cleaved in the end-standing loop (Fig. 2B). As expected, the preferred substrate of RNase T1 was the accessible G62 residue. The poorest substrate was G47, which is predicted to close the loop (Fig. 2C). Further prominent cleavage sites for RNase T1 were identified for G18, G19, and G81, supporting the predicted structure.

### Identification of targets by CopraRNA.

**FIG 3:**
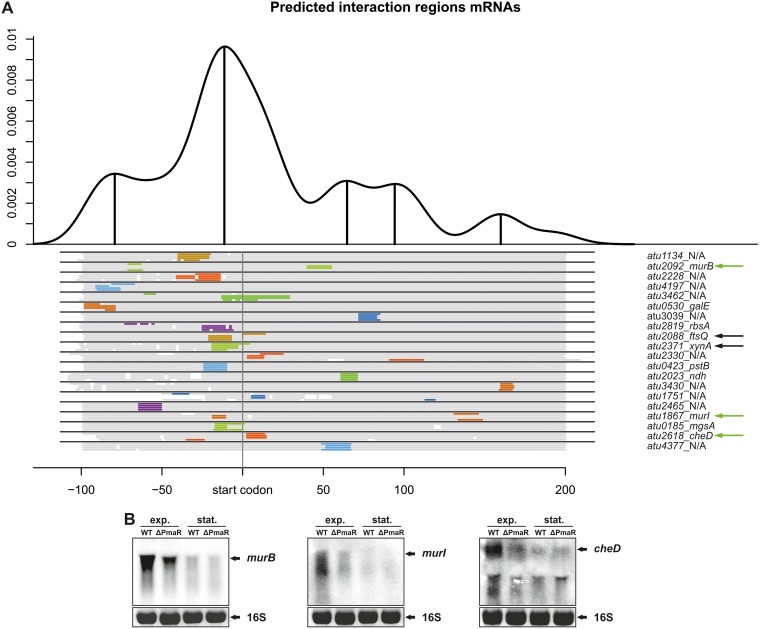
Target identification by bioinformatic prediction. (A) Interaction regions for putative target mRNAs of PmaR predicted by CopraRNA (34, 36). Prediction was performed for PmaR in *A. tumefaciens* C58 and homologues in *Rhizobium* sp. strain IRBG74, *A. tumefaciens* LBA4213, and *Agrobacterium* sp. strain H13-3. The density plot at the top shows the relative frequency of a specific mRNA nucleotide position in predicted sRNA-mRNA interactions and combines all predictions with a *P* value of ≤0.01. Local maxima are indicated with marked upright lines and indicate distinct interaction domains in the overall mRNA sequence. Below the plot, schematic alignments of the top 20 target mRNAs for all four organisms are given. Aligned regions are marked in gray, while predicted interaction regions are indicated with colors. The nomenclature of the mRNAs is presented on the right. Green arrows indicate validated targets, whereas black arrows represent targets that were tested but showed no regulation by PmaR on an RNA level. (B) Determination of mRNA transcripts by Northern blot analysis in *A. tumefaciens* wild type and ΔPmaR mutant. Strains were grown in YEB medium to exponential (exp.) and early stationary (stat.) phase and transcript amounts of *murB*, *murI*, and *cheD* were detected using specific RNA probes. Ethidium bromide-stained 16S rRNA served as a loading control.

PmaR is restricted to Agrobacterium species and Rhizobium sp. strain IRBG74, and both the sequence and structure of the sRNA are highly conserved (see Fig. S1B in the supplemental material). The target prediction tool CopraRNA (34, 36) generated a list with top candidates that are involved in peptidoglycan biosynthesis (*murB* and *murI*) and cell division (*ftsQ*) (Fig. 3A). We analyzed the transcript levels of these candidates in the presence or absence of PmaR by Northern blot analysis and found that *murB* and *murI* were both downregulated in the ΔPmaR mutant strain during exponential phase (Fig. 3B). The already low expression of these genes in early stationary phase was not further affected. PmaR had no influence on *ftsQ* mRNA amounts, and the same was true for the candidate *xynA* encoding a beta-xylanase (data not shown). Transcript levels of the candidate *cheD* encoding a methyl-accepting chemotaxis protein were downregulated in the ΔPmaR mutant during exponential phase (Fig. 3B). These results suggest that PmaR is involved in positive regulation of peptidoglycan biosynthesis, motility, and chemotaxis. Furthermore, prediction of the sRNA interaction region by CopraRNA strongly supported our hypothesis that the single-stranded loop of PmaR is responsible for target binding (Fig. S1A).

10.1128/mBio.02100-18.1 FIG S1 Bioinformatic prediction of PmaR interaction regions. (A) Interaction regions for PmaR predicted by CopraRNA. Prediction was performed for PmaR in *A. tumefaciens* C58 and homologues in *Rhizobium* sp. IRBG74, *A. tumefaciens* LBA4213, and *Agrobacterium* sp. H13-3. The density plot at the top shows the relative frequency of a specific sRNA nucleotide position in predicted sRNA-mRNA interactions and combines all predictions with a *P* value of ≤0.01. Local maxima are indicated with marked upright lines and indicate distinct interaction domains in the overall sRNA sequence. Below the plot, schematic alignments of the top 20 target mRNAs for all four organisms are given. Aligned regions are marked in gray, whereas predicted interaction regions are indicated with colors. The nomenclature of the mRNAs is presented on the right. (B) Alignment of PmaR sequences from *A. tumefaciens* C58 and homologues in *Rhizobium* sp. IRBG74, *A. tumefaciens* LBA4213, and *Agrobacterium* sp. H13-3. Conservation of the loop is indicated in blue. Download FIG S1, TIF file, 92.6 MB. Copyright © 2018 Borgmann et al. 2018 Borgmann et al. This content is distributed under the terms of the Creative Commons Attribution 4.0 International license.

### Impact of PmaR on Agrobacterium physiology.

**FIG 4:**
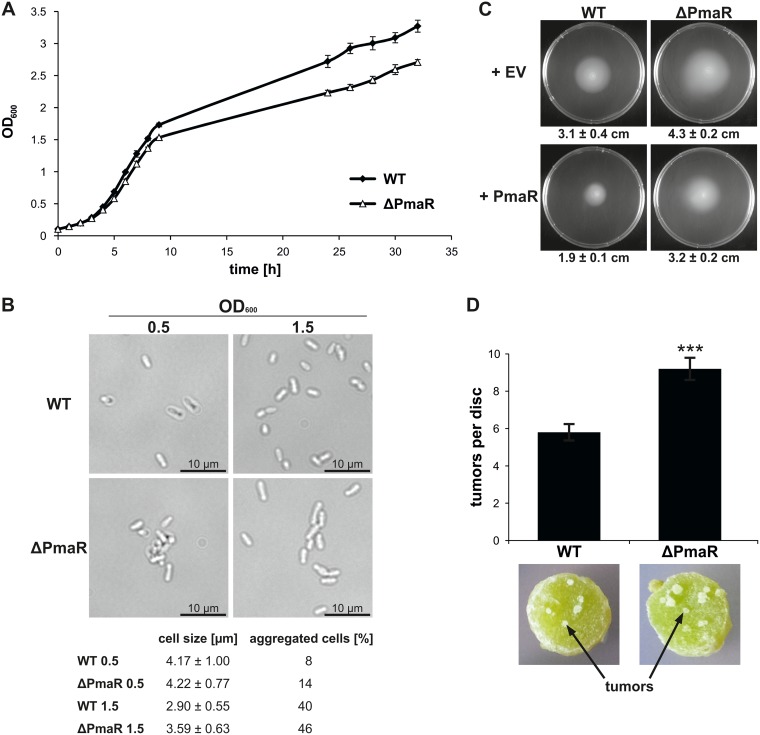
Impact of PmaR on growth, motility, and virulence. (A) Growth curve of wild type and ΔPmaR mutant grown in YEB medium. (B) Samples were taken from cultures grown in YEB medium during exponential and early stationary phase and examined by bright-field microscopy. Cell size and aggregation of 100 cells per strain were measured. (C) Motility of *A. tumefaciens* wild type and ΔPmaR mutant, both supplemented with empty vector control (+EV) and plasmid-derived PmaR (+PmaR), was determined by soft agar plates with AB medium (pH 5.5). Average motility and mean standard deviation are indicated below the pictures. (D) Potato disc infection assay with wild type and ΔPmaR mutant. Developed tumors appear white on the greenish potato discs. Experiments were performed in triplicates with similar results.

To correlate the influence of PmaR on the targets described above with Agrobacterium physiology, we tested for phenotypes of the deletion mutant. Growth of the ΔPmaR mutant strain in complex medium was slightly delayed in comparison to the wild type (Fig. 4A). Furthermore, mutant cells were slightly elongated in stationary phase and tended to aggregate in both exponential and stationary phase (Fig. 4B). Motility assays on soft agar plates revealed enhanced motility of the mutant compared to the wild type (Fig. 4C). Complementation by plasmid-borne PmaR reduced motility to wild-type values, and overexpression of the sRNA in the wild-type background almost completely abolished motility. Virulence assays revealed that the sRNA mutant elicits an increased number of tumors on potato discs (Fig. 4D). These pleiotropic effects of PmaR deletion on A. tumefaciens physiology suggest a major regulatory impact of the sRNA and prompted us to experimentally search for more targets.

### Identification of targets by mass spectrometry.

To identify further targets of PmaR, we chose a gel-free mass spectrometry approach to compare the wild-type and mutant proteomes. Since PmaR expression is induced in stationary phase (Fig. 1C), we used samples obtained from early stationary phase (optical density at 600 nm [OD_600_], 1.5). At least 10 proteins showed differential accumulation between the wild-type and ΔPmaR mutant strains. Seven of these putative targets were upregulated or exclusively detected in the ΔPmaR mutant, while three were downregulated in the mutant or exclusively found in wild-type samples (Table 1). Consistent with comparative transcript levels between the wild type and ΔPmaR mutant in stationary phase (Fig. 3B), MurB, MurI, and CheD were not found to be regulated using the proteomics approach.

**TABLE 1:** Putative targets identified by mass spectrometry

Protein	Annotated function	Regulation^*a*^
Atu1710	Conserved hypothetical protein	1.756
Atu1883	Conserved hypothetical protein	ΔPmaR mutant only
**MinD (Atu3248)**	**Cell division inhibitor**	**ΔPmaR mutant only**
**Atu3504**	**ABC transporter substrate (sulfate) binding protein**	**ΔPmaR mutant only**
Htp (Atu3604)	Hypoxanthine phosphoribosyltransferase	ΔPmaR mutant only
**PepF (Atu3765)**	**Oligoendopeptidase F**	**ΔPmaR mutant only**
Atu6048	Conserved hypothetical protein	ΔPmaR mutant only
**AmpC (Atu3077)**	**Beta-lactamase**	**0.259**
**BioB (Atu3997)**	**Biotin synthetase**	**0.622**
**BioA (Atu4000)**	**Adenosylmethionine-8-amino-7-oxononanoate aminotransferase**	**WT only**

a Regulation indicates detection of the protein either in wild type (WT) or ΔPmaR mutant only or shows the ratio between the ΔPmaR mutant and WT. Tested and validated targets on an RNA level are indicated in bold.

**FIG 5:**
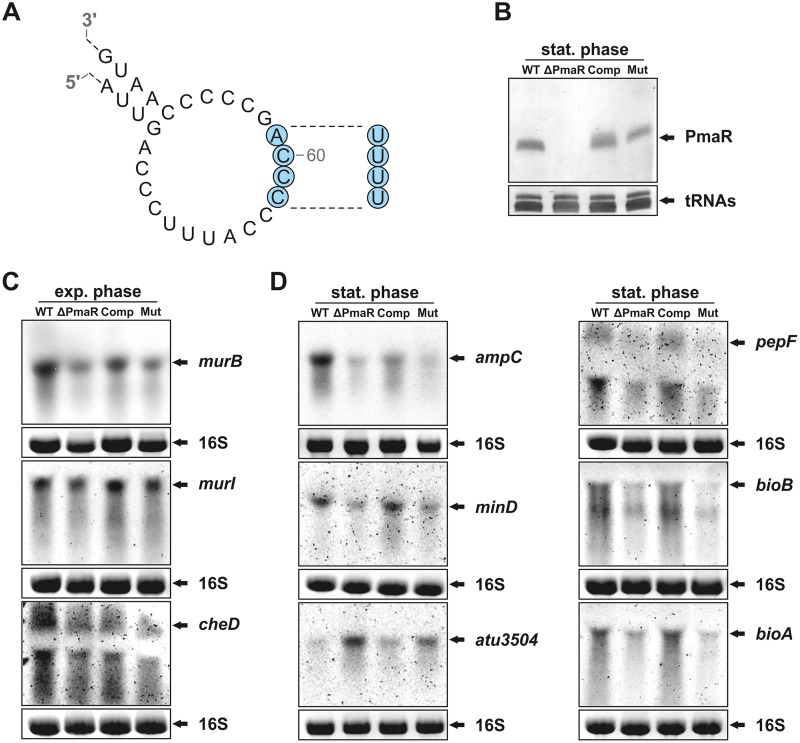
Nucleotide exchanges in the loop affect target regulation. (A) Schematic drawing of the mutated PmaR variant with four exchanged nucleotides (marked in blue). (B) Northern blot analysis of PmaR from stationary phase. Transcript levels were determined in the wild type and ΔPmaR mutant supplemented with an empty vector as well as in ΔPmaR mutant complemented with plasmid-derived wild-type PmaR (Comp) and the mutated variant (Mut). (C and D) Northern blot analysis of targets from exponential (C) and early stationary (D) phase. Transcript levels were determined in the wild type and different ΔPmaR mutant strains as described above. Ethidium bromide-stained 16S rRNA served as a loading control.

We chose six of the 10 putative targets (marked in bold in Table 1) on the basis of their annotated functions. Given the high ampicillin resistance of A. tumefaciens, one of the most interesting candidates was the beta-lactamase AmpC, the levels of which were 4-fold decreased in the absence of the sRNA. Other candidates are BioA and BioB, which are encoded in an operon and involved in biotin synthesis, and Atu3504, a substrate-binding protein of an ABC transporter of unknown function. Northern blot experiments demonstrated that the transcripts of all six targets identified by comparative proteomics were influenced by PmaR (Fig. 5D). Surprisingly, transcript levels of *minD* and *pepF* were downregulated in the ΔPmaR mutant, while the mass spectrometry data suggested upregulation of these targets in the PmaR mutant.

### Exchange of four nucleotides in the PmaR loop abolishes target regulation.

To experimentally validate the prediction that PmaR binds its targets through C-rich regions in the single-stranded loop, we designed a mutated variant with an exchange of four nucleotides (58 to 61 [CCCA-to-UUUU]; Fig. 5A) that did not alter the secondary structure of the sRNA (see probing experiments in Fig. S2) and complemented the ΔPmaR mutant with this variant. The transcript amounts of PmaR were not affected by this mutation, and the plasmid-derived sRNA variants accumulated similarly to the endogenous wild-type PmaR (Fig. 5B). Target mRNA levels in exponential and stationary phase were compared by Northern blot analysis in the wild type and mutant (with an empty vector), as well as in the complemented ΔPmaR mutant strain with wild-type sRNA (Comp) and mutated variant (Mut) (Fig. 5C and D). The three targets *murB*, *murI*, and *cheD* exhibited similar transcript patterns in exponential phase, as shown in Fig. 3B. In comparison to the strains without an empty vector (Fig. 3B), the transcript amounts of *murI* and *cheD* differed only slightly between the wild type and ΔPmaR mutant (Fig. 5C). Complementation of the ΔPmaR mutant with the wild-type sRNA restored elevated target mRNA levels, suggesting positive regulation of these targets by the sRNA. Consistent with an interaction via the C-rich motif around nucleotide 60, the target mRNA amounts in the ΔPmaR mutant did not change in the presence of the Mut plasmid (Fig. 5C). The same pattern was observed for the target mRNAs *ampC*, *minD*, *pepF*, *bioA*, and *bioB* in stationary phase, indicating positive regulation by the same sRNA region. As suggested by the proteomics data (Table 1) and in accordance with negative regulation by PmaR, *atu3504* showed the opposite transcript pattern (Fig. 5D). The mRNA levels were low in the wild type and Comp strains but elevated in the ΔPmaR mutant and the complementation with the Mut plasmid, which shows that the same sRNA region is involved in positive and negative regulation.

10.1128/mBio.02100-18.2 FIG S2 Secondary structure of the mutated PmaR variant. (A) Predicted secondary structure of PmaR Mut by RNAfold (Institute for Theoretical Chemistry, University of Vienna [http://rna.tbi.univie.ac.at/]). Nucleotide exchanges in the loop are marked in blue. Arrows indicate cleavage sites for RNases T1 and T2, respectively. (B) Enzymatic structure probing of PmaR Mut with RNases T1 and T2 and nuclease S1. RNA treated with water (first lane) served as a control. L_OH_, alkaline ladder. Download FIG S2, TIF file, 76.3 MB. Copyright © 2018 Borgmann et al. 2018 Borgmann et al. This content is distributed under the terms of the Creative Commons Attribution 4.0 International license.

**FIG 6:**
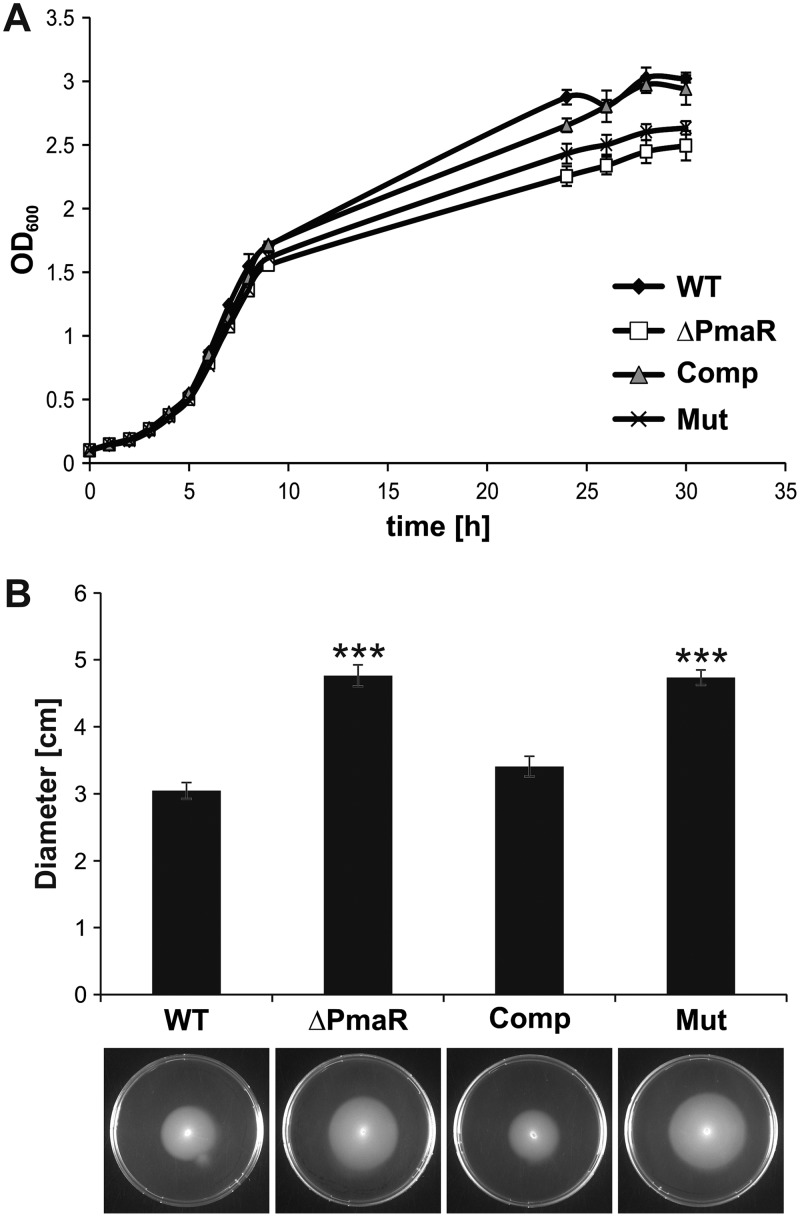
Mutated PmaR variant cannot restore wild-type growth and motility. (A) Growth curves in YEB medium of wild type and ΔPmaR mutant supplemented with an empty vector and ΔPmaR mutant complemented with plasmid-derived wild-type PmaR (Comp) and the mutated variant (Mut). (B) Motility of the four *A. tumefaciens* strains on soft agar plates with AB medium (pH 5.5). Experiments were performed in triplicate with similar results.

The importance of the exchanged nucleotides was further corroborated by growth experiments (Fig. 6A) and motility assays (Fig. 6B) of the complemented mutant strain. Complementation with wild-type PmaR restored wild-type growth and motility, whereas complementation with the mutated variant resulted in reduced growth and enhanced motility comparable to those of the PmaR mutant. Taken together, these data confirm that the four mutated nucleotides in the sRNA loop are essential for target mRNA regulation.

### Regulation of target mRNAs *in vivo*.

**FIG 7:**
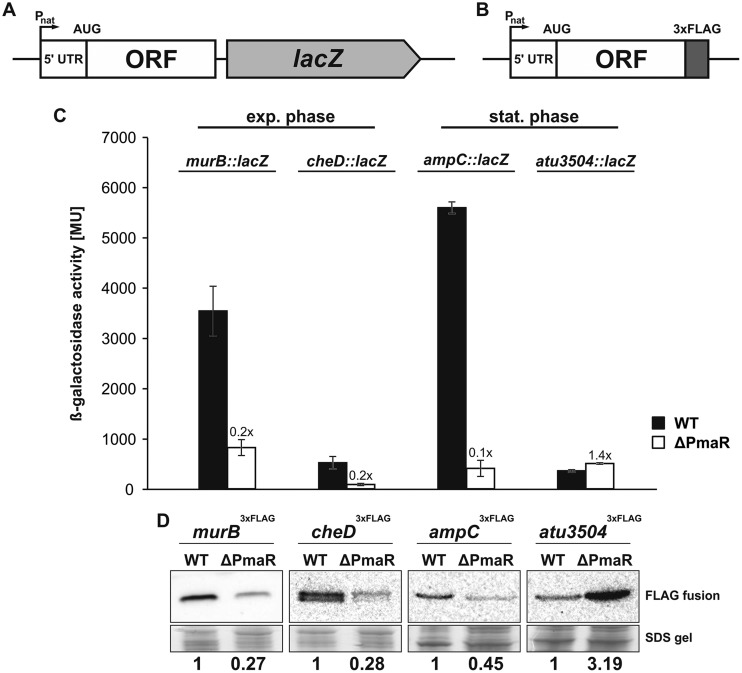
Impact of PmaR on targets *in vivo*. Schematic drawing of transcriptional *lacZ* fusions (A) and translational fusions with 3×FLAG sequence (B). Reporter constructs were integrated into the chromosome of wild type and ΔPmaR mutant and expressed from the native promoter (P_nat_). (C) Expression of targets in wild type (black bars) and ΔPmaR mutant (white bars) quantified by β-galactosidase activity (in Miller units) of transcriptional *lacZ* fusions. Mean standard deviation and induction rates relative to the wild type are indicated. Experiments were performed in triplicate, with three replicates each. (D) Western blot analysis of translational target fusions in wild type and ΔPmaR mutant via anti-3×FLAG M2 antibody. Quantification of detected signals was performed by pixel counting, and Coomassie-stained SDS-gels served as loading control. Experiments were performed in triplicate, with similar results. ORF, open reading frame.

In order to monitor target regulation by PmaR *in vivo*, we constructed transcriptional (Fig. 7A) and translational (Fig. 7B) reporter fusions of targets in exponential (*murB* and *cheD*) or stationary phase (*ampC* and *atu3504*) and introduced them into the chromosome of the Agrobacterium wild type and the ΔPmaR mutant under the control of the native promoter. Transcript levels were measured with a *lacZ* fusion via β-galactosidase activity assays, and protein levels were monitored by Western blot analysis detecting a fused 3×FLAG epitope. Fully consistent with the assumed positive regulation, the transcript levels of *murB*, *cheD*, and *ampC* were strongly reduced in the ΔPmaR mutant in comparison to the wild type (Fig. 7C). The response on an RNA level was mirrored by a reduction in the corresponding protein amounts (Fig. 7D). In accordance with negative regulation by the sRNA, *atu3504* mRNA levels (Fig. 7C) and, in particular, Atu3504 protein levels (Fig. 7D), were induced in the ΔPmaR mutant.

**FIG 8:**
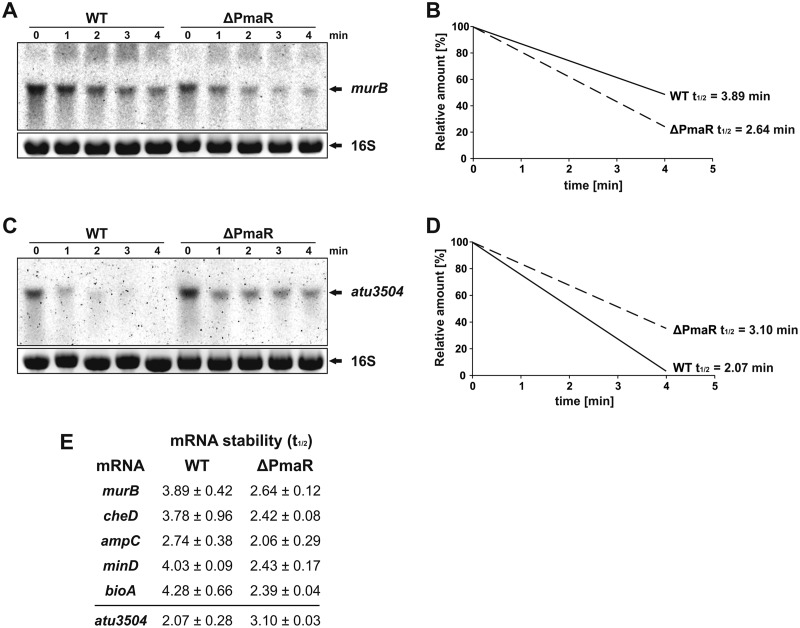
PmaR influences transcript stability of target mRNAs. Northern blot analysis of *murB* (A) and *atu3504* (C) in wild type and the ΔPmaR mutant after the addition of rifampin. Ethidium bromide-stained 16S rRNA served as loading control. Transcript half-lives (*t*_1/2_) of *murB* (B) and *atu3504* (D) were calculated by pixel counting from three biological replicates. (E) Transcript stability of different target mRNAs in wild type and ΔPmaR mutant. Half-lives were calculated from at least two biological replicates.

Since both the transcript and protein levels of the examined fusions were altered in the ΔPmaR mutant, we assumed that PmaR might affect the stability of target mRNAs. We measured the half-lives of PmaR targets by adding rifampin to wild-type and ΔPmaR mutant cultures and taking samples before and up to 4 min after treatment with the transcription inhibitor. Northern blot analysis confirmed that transcript stability of the positively regulated *murB* transcript was decreased in the ΔPmaR mutant (Fig. 8A and B), whereas the stability of the negatively regulated *atu3504* mRNA was elevated in the PmaR mutant (Fig. 8C and D). Consistent with the observed downregulation in the ΔPmaR mutant (Fig. 5C and D), the transcript stability of the PmaR targets *cheD, ampC*, *minD,* and *bioA* was decreased in the ΔPmaR mutant strain (Fig. 5E).

### Direct binding of target mRNAs by PmaR *in vitro*.

**FIG 9:**
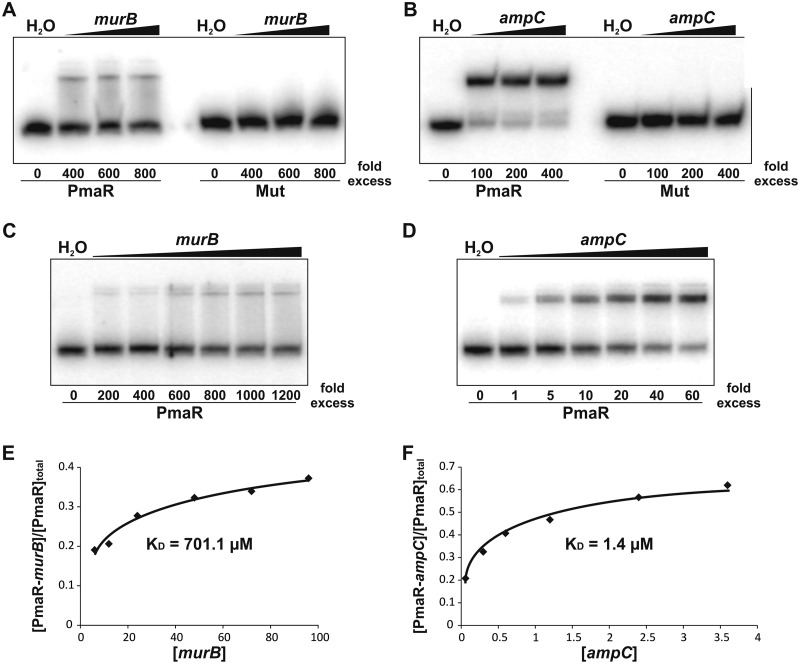
PmaR interacts with *murB* and *ampC in vitro*. (A and B) Electrophoretic mobility shift assays for *murB* (A) and *ampC* (B) with labeled PmaR and the mutated variant (Mut). Final concentrations of unlabeled RNA were added in 400- to 800-fold excess for *murB* and in 100- to 400-fold excess for *ampC*. Samples treated with water served as a control. (C and D) Concentration series for *murB* (C) and *ampC* (D) with labeled wild-type PmaR. Samples treated with water served as a control. (E and F) Binding kinetics and calculated *K_D_* values for complex formation of *murB* (E) and *ampC* (F) with PmaR by pixel counting of panels C and D. Experiments were performed in triplicate, with similar results.

The CopraRNA program predicted sRNA-mRNA interactions in various regions of the PmaR targets (Fig. 3A). We chose *murB* and *ampC,* which are both predicted to bind PmaR in their 5′ untranslated region (UTR) (Fig. S3A and S4A) for monitoring a direct interaction with PmaR by electrophoretic mobility shift assays. Both targets bound PmaR resulting in a gel shift, whereas the mutated PmaR variant with the exchanged CCCA region did not produce a shift (Fig. 9A and B). Remarkably, the binding affinities of *murB* and *ampC* to PmaR were vastly different. A hundredfold excess of *murB* was necessary to retard wild-type PmaR (Fig. 9C), whereas equimolar amounts of *ampC* were sufficient to shift the sRNA (Fig. 9D). We quantified the signals of Fig. 9C and D and plotted the binding affinity of both *murB* (Fig. 9E) and *ampC* (Fig. 9F) to PmaR. The calculated *K_D_* (equilibrium dissociation constant) values for *murB* and *ampC* were around 700 and 1 μM, respectively (Fig. 9E and F).

10.1128/mBio.02100-18.3 FIG S3 Prediction of interaction regions with targets from exponential phase. Predicted interaction regions of PmaR with *murB* (A), *murI* (B), and *cheD* (C) by IntaRNA. The calculated energy score of each interaction is indicated below the interaction schemata. Download FIG S3, TIF file, 84.3 MB. Copyright © 2018 Borgmann et al. 2018 Borgmann et al. This content is distributed under the terms of the Creative Commons Attribution 4.0 International license.

10.1128/mBio.02100-18.4 FIG S4 Prediction of interaction regions with targets from stationary phase. Predicted interaction regions of PmaR with *ampC* (A), *minD* (B), *atu3504* (C), *pepF* (D), *bioB* (E), and *bioA* (F) by IntaRNA. The calculated energy score of each interaction is indicated below the interaction schemata. Download FIG S4, TIF file, 95.6 MB. Copyright © 2018 Borgmann et al. 2018 Borgmann et al. This content is distributed under the terms of the Creative Commons Attribution 4.0 International license.

### PmaR positively controls ampicillin resistance.

**FIG 10:**
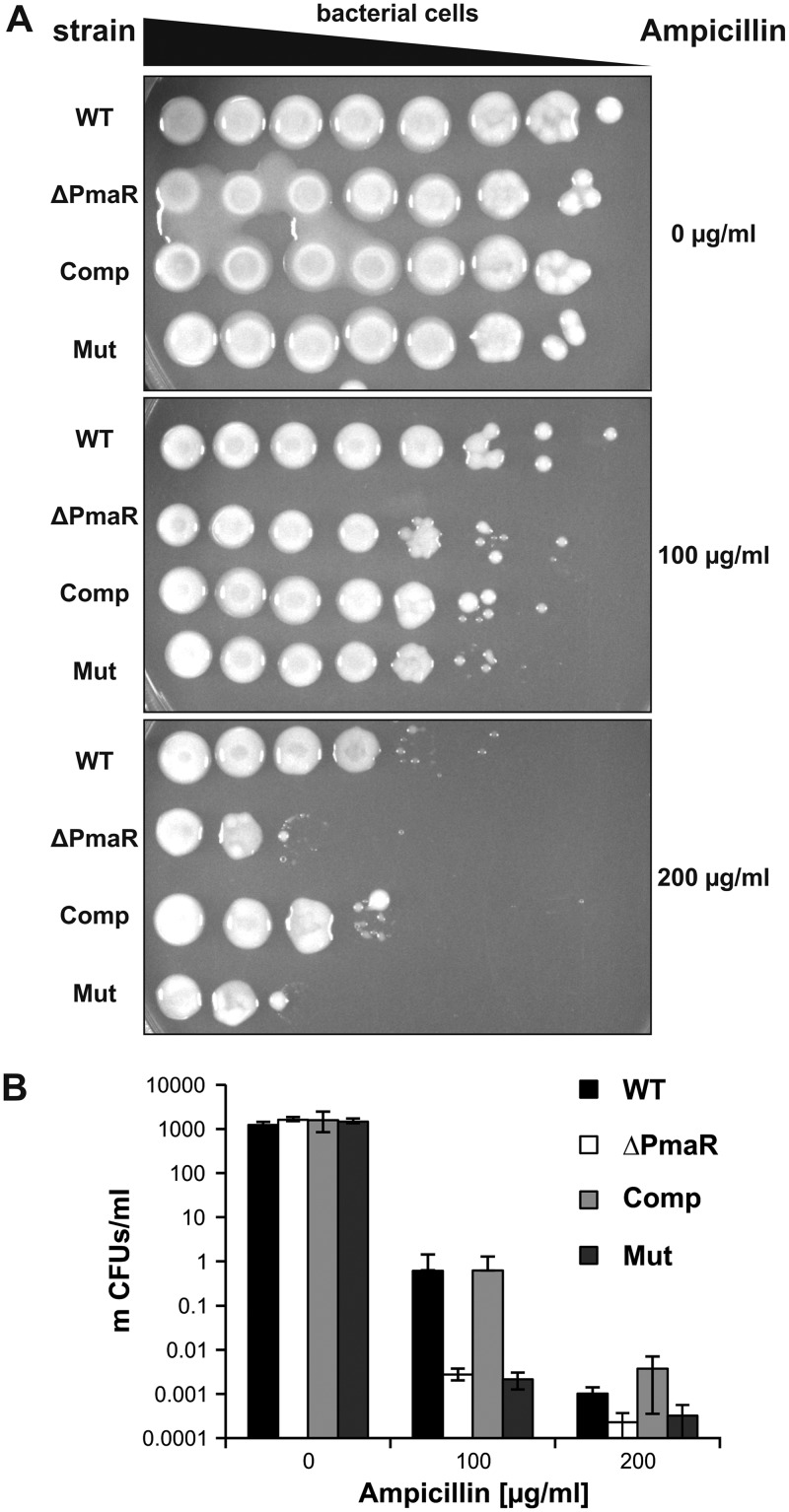
Regulation of ampicillin resistance by PmaR. Serial dilutions of cultures from wild type and ΔPmaR mutant supplemented with an empty vector as well as ΔPmaR mutant complemented with plasmid-derived wild-type PmaR (Comp) and the mutated variant (Mut) were spotted (A) and plated (B) on YEB medium with different concentrations of ampicillin. CFU were counted and calculated per milliliter of culture. Experiments were performed in triplicate, with similar results.

The beta-lactamase AmpC is highly conserved among *Rhizobiaceae* and confers high resistance to β-lactam antibiotics, such as ampicillin. Direct interaction of PmaR with the *ampC* transcript *in vitro,* as well as decreased *ampC* mRNA and AmpC protein levels in the PmaR mutant *in vivo,* strongly suggest a direct regulation of ampicillin resistance by the sRNA in A. tumefaciens. Hence, we examined the sensitivity of the PmaR mutant to ampicillin. The sRNA mutant indeed displayed higher sensitivity to the antibiotic at a concentration of 200 µg/ml than did the wild type (Fig. 10A and B). Moreover, complementation of the mutant with wild-type (WT) PmaR resulted in WT-like resistance to ampicillin. The sRNA deletion strain complemented with the Mut plasmid remained sensitive to the antibiotic. Sensitivity to the carboxypenicillin ticarcillin, which is widely used in plant biotechnology to kill A. tumefaciens after plant infection, was not affected by PmaR (data not shown).

## DISCUSSION

### Deletion of PmaR leads to pleiotropic effects on A. tumefaciens physiology.

**FIG 11:**
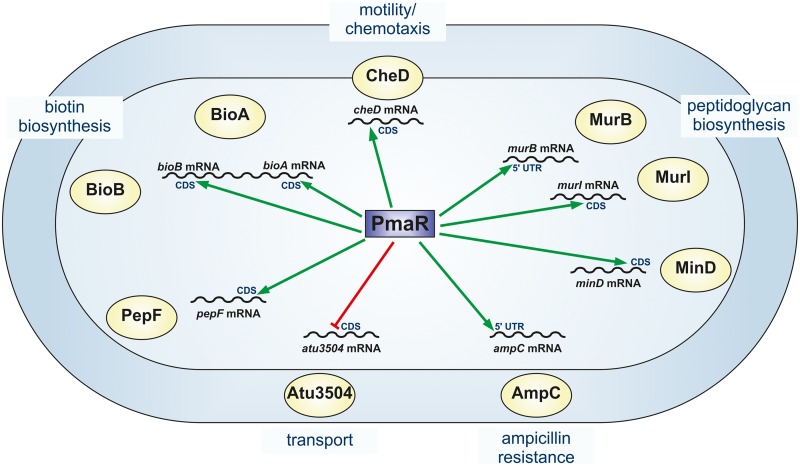
Impact of PmaR on *A. tumefaciens*. Schematic overview of PmaR functions in the cell. Positive (green) and negative (red) regulation of target mRNAs by PmaR and the predicted interaction regions in mRNA sequences (5ʹ UTR or coding sequence [CDS]) are indicated.

In this study, we examined the function of the small regulatory RNA PmaR in the plant pathogen A. tumefaciens. We discovered a broad impact of PmaR on Agrobacterium physiology, including peptidoglycan biosynthesis, motility, and virulence. Using a combination of bioinformatic predictions with CopraRNA (34, 36) and comparative proteomics by mass spectrometry, we identified nine targets that are affected by PmaR at the RNA and protein levels. Several of these targets can explain the observed phenotypes of the PmaR deletion mutant (Fig. 11). The moderate growth defect of the ΔPmaR mutant may result from decreased expression of *murB* and *murI* leading to perturbations in peptidoglycan formation. Further, decreased levels of BioA and BioB in the mutant most likely cause lower biotin levels in the cell that can reduce bacterial growth. Biotin is important for fatty acid biosynthesis, and decreased biotin levels in the cell can cause a severe imbalance in the bacterial cell envelope. Already in 1933, biotin was described to enhance the growth of rhizobial isolates (37, 38). Although A. tumefaciens is able to synthesize profligate amounts of the vitamin, which might be beneficial in its ecological niche, biotin is needed for growth in minimal medium (39).

Regulation of the target *cheD* by PmaR is in accordance with the observed motility phenotype. PmaR-dependent regulation of *cheD* was dependent on the growth phase, and slightly enhanced *cheD* levels were found in ΔPmaR mutant during stationary phase (Fig. 3B). This regulation may well reflect the conditions during the motility assay where the cells reside in stationary phase after 48 h of incubation. Another interesting observation is the enhanced tumor formation of the PmaR mutant. We did not identify any direct virulence-related target of the sRNA and propose that the enhanced tumor formation is due to a secondary effect, for example, the enhanced motility of ΔPmaR mutant promoting a more efficient infection of the potato discs. To the best of our knowledge, the only other previously described hypervirulent mutant of A. tumefaciens is impaired in the formation of ornithine lipids (40). In that mutant, the causal relationship between this process and tumorigenesis is not yet clear.

Small RNAs often negatively regulate ABC transporters, and a prominent example in alphaproteobacteria is the AbcR family (41). Based on the downregulation of *atu3504*, PmaR may have a related function in nutrient acquisition. Atu3504 is annotated as an ABC transporter substrate-binding protein for sulfate. Therefore, higher levels of this protein in the ΔPmaR mutant might result in more efficient sulfate uptake, although we found that growth of the wild type and the PmaR mutant was identical under sulfate-limiting conditions in minimal medium (data not shown). This can be attributed to multiple alternative sulfate transporters or an inaccurate annotation of Atu3504. At any rate, regulation of this protein follows the common trend that sRNAs downregulate substrate-binding proteins.

Several sRNAs influence bacterial resistance to antibiotics by their impact on the biosynthesis of porins, efflux systems, or biofilm formation (9). We provide complementary evidence *in vivo* and *in vitro* that PmaR is an sRNA that directly controls a bacterial antibiotic resistance gene coding for the beta-lactamase AmpC, which is highly conserved in *Rhizobiaceae* (24, 25). PmaR is only present in Agrobacterium species and a closely related Rhizobium strain. Due to their short sequence, sRNAs are known to evolve rapidly. The even shorter seed sequences that are sufficient for target interaction allow an enhanced turnover of sRNAs in terms of their *de novo* emergence, frequent change of function, or loss from bacterial lineages (42, 43). It is possible that PmaR is a recently evolved feature of Agrobacterium to stabilize *ampC* transcripts, thereby ensuring sufficient amounts of beta-lactamase. This may provide a competitive advantage of A. tumefaciens in the microbe-rich environment of the rhizosphere.

### PmaR influences transcript stability and translation.

In contrast to the majority of previously studied sRNAs (44), PmaR positively regulates most of its targets. Not surprisingly, the interacting sequence of PmaR does not consist of an anti-Shine-Dalgarno region as in AbcR1 and many other sRNAs that regulate translation initiation (32), but it consists primarily of cytosine residues. Most of the predicted interaction regions of PmaR in the target mRNAs are not located close to the ribosome binding site or the start codon. Instead, the program CopraRNA (34, 36) predicted various interaction regions throughout the transcripts (Fig. 3A). Predictions with IntaRNA (36, 45, 46) for the nine experimentally validated targets suggested interactions both in the 5′UTR (*murB* and *ampC*) and the coding sequence (Fig. S3 and S4). Prediction of the secondary structures of *murB* and *ampC* by Mfold (47) revealed structures that might prevent ribosome binding due to base pairing of the Shine-Dalgarno sequence (data not shown). Structural rearrangement of the 5′UTRs upon binding of PmaR might facilitate ribosome binding and therefore promote translation and/or stabilize the mRNAs as shown in various other cases (48). A similar mode of action might apply to *cheD,* since the interaction site was predicted immediately downstream of the start codon. These hypotheses are supported by the observed decrease in *murB* and *cheD* transcript stability in the ΔPmaR mutant (Fig. 8A, B, and E). For the other target mRNAs, we suggest that the binding of PmaR might either block RNase cleavage sites or open complex secondary structures to allow translation of the mRNA to proceed. Interestingly, the downregulated target *atu3504* also does not conform to the standard mechanism, in which an sRNA binds at or around the Shine-Dalgarno region. Instead, the predicted sRNA-mRNA interaction site is far within the coding sequence (Fig. S4C), and PmaR seems to destabilize the *atu3504* transcript (Fig. 8C and D), suggesting that other factors are involved in the regulatory process.

### Growth phase-dependent competition of targets for PmaR binding.

PmaR controls a complex network of at least nine targets (Fig. 11). The abundance of both the sRNA and the target mRNAs changes throughout growth, suggesting that the relative sRNA-mRNA concentrations vary constantly and that the targets compete for binding of the sRNA. Despite low expression of PmaR in exponential phase, it regulates several targets under this condition. Other targets are primarily regulated in stationary phase. Interestingly, the affinities of PmaR to *murB* (regulated in exponential phase) and *ampC* (regulated in stationary phase) are very different. However, the calculated dissociation constants are derived from *in vitro* experiments and may not reflect the actual conditions inside the cell, where mRNAs are probably targeted by several different sRNAs and RNA-binding proteins mediate sRNA-mRNA interactions. From a “target-centric” perspective, it is possible that targets in stationary phase, such as *ampC,* compete for PmaR binding, neutralizing the effect of the sRNA on targets from exponential phase, as reviewed previously (1, 49). The band shift results contradict the bioinformatic predictions by IntaRNA (36, 45, 46) that proposed stronger binding of PmaR to *murB* (Fig. S3A) than to *ampC* (Fig. S4A) and show how misleading predictions without experimental validation can be. Overall, our data suggest that under specific conditions, competition of target mRNAs for PmaR binding determines the sRNA function rather than the mere expression of the sRNA.

## MATERIALS AND METHODS

### Bacterial growth conditions.

The bacterial strains used in this study are listed in Table S1. A. tumefaciens C58 was cultivated in yeast extract-beef extract (YEB) complex medium at 30°C to OD_600_s of 0.4 (exponential phase) and 1.5 (early stationary phase), respectively. Cultivation in minimal medium (AB) and subsequent virulence induction were performed as described previously (29). E. coli was grown in LB medium at 37°C. Media were supplemented with ampicillin (Amp, 100 µg/ml) or kanamycin (50 µg/ml) if required.

10.1128/mBio.02100-18.5 TABLE S1 Strains and plasmids used in this study. Download Table S1, DOCX file, 0.02 MB. Copyright © 2018 Borgmann et al. 2018 Borgmann et al. This content is distributed under the terms of the Creative Commons Attribution 4.0 International license.

Ampicillin sensitivity assays were performed by cultivation of A. tumefaciens strains to an OD_600_ of 1.0 (10^9^ cells). Serial dilutions in A. dest buffer were spotted or plated on YEB medium with or without ampicillin and incubated at 30°C.

### Strain and vector construction.

The oligonucleotides and plasmids used in this study are listed in Tables S1 and S2, respectively.

10.1128/mBio.02100-18.6 TABLE S2 Oligonucleotides used in this study. Restriction sites are underlined, and T7 promoter sequences for generation of *in vitro* transcripts are in bold letters. Exchanged nucleotides used for site-directed mutagenesis are marked in gray. Download Table S2, DOCX file, 0.02 MB. Copyright © 2018 Borgmann et al. 2018 Borgmann et al. This content is distributed under the terms of the Creative Commons Attribution 4.0 International license.

Deletion of PmaR was performed as described previously (32, 50). Complementation of the ΔPmaR mutant was achieved by cloning PmaR into pSRK, as described previously (51), and the resulting construct was introduced into A. tumefaciens by electroporation.

Transcriptional *lacZ* reporter gene fusions were constructed by amplifying the complete open reading frame of a target gene plus 75 nt of the 5ʹ UTR by PCR using the corresponding primer pairs (Table S2). The fragments were inserted blunt end into pUC19. A *lacZ*-Gm^r^-*oriT* cassette derived from pYP141I (Y. Pfänder and B. Masepohl, unpublished data) was introduced in these pUC19 constructs via the primer-derived BamHI restriction site that was added to the 3ʹ end of the target genes. The resulting reporter constructs were introduced into the chromosome of the A. tumefaciens wild type and ΔPmaR mutant by single-crossover integration via electroporation and expressed from the native promoter.

Translational reporter gene fusions were constructed by insertion of target gene sequences without the stop codon into pUC19, as described above. A 3×FLAG-Km^r^-*oriT* cassette derived from pYP247I (52) was introduced in these pUC19 constructs via an SmaI restriction site. The resulting reporter constructs were introduced into the chromosome of A. tumefaciens, as described above.

Runoff plasmids for *in vitro* transcriptions were constructed by amplifying specific target gene sequences (150 nt) by PCR using the corresponding primer pairs (Table S2) and ligation into pUC19. A primer-derived T7 promoter sequence was added to the 5ʹ end, while an EcoRV restriction site was added to the 3ʹ end of the target gene sequence.

Site-directed mutagenesis of PmaR in pSRK or runoff plasmids was achieved using the corresponding primer pairs (Table S2).

### Motility test.

Determination of motility was performed by spotting 3 µl of liquid overnight cultures on soft agar plates containing AB minimal medium (pH 5.5) with an agar concentration of 0.5% (wt/vol). Plates were incubated at 30°C for 48 h.

### Potato disc infection assays.

Quantitative virulence assays with potato tuber discs were carried out as described previously (53).

### β-Galactosidase activity assay.

A. tumefaciens strains harboring chromosomal 3×FLAG fusions to target genes were grown at 30°C in YEB medium to indicated growth phases. Cells (1 ml) were harvested by centrifugation and resuspended in 800 µl of 10× Z-buffer. Cells were permeabilized with chloroform and 0.01% SDS. Enzymatic reactions were started by adding 200 µl *o*-nitrophenyl-β-d-galactopyranoside (ONPG) (4 mg/ml) and stopped by adding 500 µl Na_2_CO_3_. *o*-Nitrophenyl (ONP) production at 420 nm was measured.

### Western blot analysis.

Protein samples were separated via SDS-PAGE (12%) gels and subsequently transferred onto nitrocellulose membranes (Hybond-C Extra; GE Healthcare, Munich, Germany) by tank blotting. Anti-3×FLAG M2 antibody (Sigma-Aldrich, Germany) and secondary goat anti-mouse horseradish peroxidase (HRP) conjugate (Bio-Rad, Munich, Germany) were used in a 1:5,000 dilution. Detection by luminescence was performed using Luminata Forte Western HRP substrate (Merck, Darmstadt, Germany) and the Chemi Imager Ready system (Alpha Innotec, San Leandro, CA, USA).

### Identification of target mRNAs by mass spectrometry.

Tryptic digestion, mass spectrometry, and data processing for proteomic profiling were essentially performed as described previously (54), with the following changes: mass range, *m/z* 50 to 1,200, and scan time, 1 s/scan. For data processing using ProteinLynx Global Server (version 2.5.2; Waters), a nonredundant version of the A. tumefaciens (BioProject PRJNA57865) database containing 5,558 protein entries (including sequences for rabbit PhosB quantitation standard [Waters], trypsin, and keratin) was used for protein identification. Proteins were considered up- or downregulated when they were identified (i) in all three biological replicates in the mutant samples but not in the wild-type samples or vice versa, or (ii) in at least two of three biological replicates with *P* values below 0.05 and with the following ratios exceeding a threshold of 0.653/1.540. Thresholds were calculated using a confidence interval of 95% (mean ratio ± 1.96 × standard deviation).

### RNA preparation.

A. tumefaciens cells (10 ml) were harvested for RNA preparation, as described previously (32). Isolation of total RNA was performed using the hot acid phenol method (55). Stability assays were performed by cultivation of the wild type and ΔPmaR mutant to exponential or stationary phase and subsequent addition of rifampin to a final concentration of 250 µg/ml. Samples for RNA preparation were taken before and 1, 2, 3, and 4 min after rifampin treatment.

### Northern blot analysis.

PmaR transcript levels were detected by Northern blot analysis, as described before (32). Hybridization with a digoxigenin-labeled RNA probe (Roche, Mannheim, Germany) was performed at 42°C overnight. Washing steps and detection by using chemiluminescence substrate CDP-Star (Roche) were carried out as described previously (32). Detection of target mRNAs was performed using the vacuum blot technique, as described before (56).

### Enzymatic RNA structure probing.

To elucidate the RNA structure of PmaR, transcripts were synthesized *in vitro* by runoff transcription from EcoRV-linearized plasmids (listed in Table S1) with T7 RNA polymerase. The sRNA was purified, dephosphorylated with calf intestinal alkaline phosphatase (CIP; Thermo Scientific, Waltham, MA, USA), and radioactively labeled at the 5′ end, as described before (57). Partial digestions with ribonucleases T1 (0.02 U) (Thermo Scientific) and T2 (0.45 U) (MoBiTec, Göttingen, Germany) and nuclease S1 (1 U) (Thermo Scientific) were performed at 30°C, as described previously (58).

### Electrophoretic mobility shift assays.

RNA transcripts were synthesized *in vitro* by runoff transcription, as described above. RNA band shift experiments were performed in 1× structure buffer (Ambion, Austin, TX, USA) using 5,000 cpm-labeled sRNA and unlabeled *murB* and *ampC* mRNA fragments. Final concentrations of mRNA fragments are given in Fig. 8A to D. Samples were incubated in the presence of 1 µg tRNA at 30°C for 30 min. Binding reactions were stopped with 3 µl native loading dye (50% glycerol, 0.5× Tris-borate-EDTA [TBE], 0.1% bromophenol blue, and 0.1% xylene cyanol) and separated on native 6% polyacrylamide gels in 0.5× TBE at 300 V for 1 h.

## References

[1] Nitzan M, Rehani R, Margalit H 2017 Integration of bacterial small RNAs in regulatory networks. Annu Rev Biophys 46:131–148. doi:10.1146/annurev-biophys-070816-034058. 28532217

[2] Storz G, Vogel J, Wassarman KM 2011 Regulation by small RNAs in bacteria: expanding frontiers. Mol Cell 43:880–891. doi:10.1016/j.molcel.2011.08.022. 21925377 PMC3176440

[3] Lalaouna D, Simoneau-Roy M, Lafontaine D, Massé E 2013 Regulatory RNAs and target mRNA decay in prokaryotes. Biochim Biophys Acta 1829:742–747. doi:10.1016/j.bbagrm.2013.02.013. 23500183

[4] Bouche F, Bouche JP 1989 Genetic evidence that DicF, a second division inhibitor encoded by the *Escherichia coli dicB* operon, is probably RNA. Mol Microbiol 3:991–994. doi:10.1111/j.1365-2958.1989.tb00249.x. 2477663

[5] Holmqvist E, Wagner EGH 2017 Impact of bacterial sRNAs in stress responses. Biochem Soc Trans 45:1203–1212. doi:10.1042/BST20160363. 29101308 PMC5730939

[6] Papenfort K, Silpe JE, Schramma KR, Cong JP, Seyedsayamdost MR, Bassler BL 2017 A *Vibrio cholerae* autoinducer-receptor pair that controls biofilm formation. Nat Chem Biol 13:551–557. doi:10.1038/nchembio.2336. 28319101 PMC5391282

[7] Harris JF, Micheva-Viteva S, Li N, Hong-Geller E 2013 Small RNA-mediated regulation of host-pathogen interactions. Virulence 4:785–795. doi:10.4161/viru.26119. 23958954 PMC3925712

[8] Papenfort K, Vogel J 2014 Small RNA functions in carbon metabolism and virulence of enteric pathogens. Front Cell Infect Microbiol 4:91. doi:10.3389/fcimb.2014.00091. 25077072 PMC4098024

[9] Dersch P, Khan MA, Mühlen S, Görke B 2017 Roles of regulatory RNAs for antibiotic resistance in bacteria and their potential value as novel drug targets. Front Microbiol 8:803. doi:10.3389/fmicb.2017.00803. 28529506 PMC5418344

[10] Georg J, Hess WR 2011 *cis*-antisense RNA, another level of gene regulation in bacteria. Microbiol Mol Biol Rev 75:286–300. doi:10.1128/MMBR.00032-10. 21646430 PMC3122628

[11] Papenfort K, Vogel J 2009 Multiple target regulation by small noncoding RNAs rewires gene expression at the post-transcriptional level. Res Microbiol 160:278–287. doi:10.1016/j.resmic.2009.03.004. 19366629

[12] Santiago-Frangos A, Woodson SA 2018 Hfq chaperone brings speed dating to bacterial sRNA. Wiley Interdiscip Rev RNA 9:e1475. doi:10.1002/wrna.1475. 29633565 PMC6002925

[13] Gelsinger DR, DiRuggiero J 2018 The non-coding regulatory RNA revolution in Archaea. Genes 9:141. doi:10.3390/genes9030141. PMC5867862 29510582

[14] Sharma CM, Vogel J 2014 Differential RNA-seq: the approach behind and the biological insight gained. Curr Opin Microbiol 19:97–105. doi:10.1016/j.mib.2014.06.010. 25024085

[15] Mank NN, Berghoff BA, Hermanns YN, Klug G 2012 Regulation of bacterial photosynthesis genes by the small noncoding RNA PcrZ. Proc Natl Acad Sci U S A 109:16306–16311. doi:10.1073/pnas.1207067109. 22988125 PMC3479615

[16] Becker A, Overlöper A, Schlüter JP, Reinkensmeier J, Robledo M, Giegerich R, Narberhaus F, Evguenieva-Hackenberg E 2014 Riboregulation in plant-associated α-proteobacteria. RNA Biol 11:550–562. doi:10.4161/rna.29625. 25003187 PMC4152362

[17] Robledo M, Frage B, Wright PR, Becker A 2015 A stress-induced small RNA modulates alpha-rhizobial cell cycle progression. PLoS Genet 11:e1005153. doi:10.1371/journal.pgen.1005153. 25923724 PMC4414408

[18] Robledo M, Peregrina A, Millán V, García-Tomsig NI, Torres-Quesada O, Mateos PF, Becker A, Jiménez-Zurdo JI 2017 A conserved α-proteobacterial small RNA contributes to osmoadaptation and symbiotic efficiency of rhizobia on legume roots. Environ Microbiol 19:2661–2680. doi:10.1111/1462-2920.13757. 28401641

[19] Sheehan LM, Caswell CC 2017 A 6-nucleotide regulatory motif within the AbcR small RNAs of Brucella abortus mediates host-pathogen interactions. mBio 8:e00473-17. doi:10.1128/mBio.00473-17. 28588127 PMC5461406

[20] Lassalle F, Campillo T, Vial L, Baude J, Costechareyre D, Chapulliot D, Shams M, Abrouk D, Lavire C, Oger-Desfeux C, Hommais F, Gueguen L, Daubin V, Muller D, Nesme X 2011 Genomic species are ecological species as revealed by comparative genomics in *Agrobacterium tumefaciens*. Genome Biol Evol 3:762–781. doi:10.1093/gbe/evr070. 21795751 PMC3163468

[21] Barton IS, Fuqua C, Platt TG 2018 Ecological and evolutionary dynamics of a model facultative pathogen: *Agrobacterium* and crown gall disease of plants. Environ Microbiol 20:16–29. doi:10.1111/1462-2920.13976. 29105274 PMC5764771

[22] Gelvin SB 2012 Traversing the cell: *Agrobacterium* T-DNA’s journey to the host genome. Front Plant Sci 3:52. doi:10.3389/fpls.2012.00052. 22645590 PMC3355731

[23] Pitzschke A, Hirt H 2010 New insights into an old story: *Agrobacterium*-induced tumour formation in plants by plant transformation. EMBO J 29:1021–1032. doi:10.1038/emboj.2010.8. 20150897 PMC2845280

[24] Ogawa Y, Mii M 2004 Screening for highly active β-lactam antibiotics against *Agrobacterium tumefaciens*. Arch Microbiol 181:331–336. doi:10.1007/s00203-004-0650-z. 14752589

[25] Poirel L, Naas T, Nordmann P 2010 Diversity, epidemiology, and genetics of class D β-lactamases. Antimicrob Agents Chemother 54:24–38. doi:10.1128/AAC.01512-08. 19721065 PMC2798486

[26] Dequivre M, Diel B, Villard C, Sismeiro O, Durot M, Coppée JY, Nesme X, Vial L, Hommais F 2015 Small RNA deep-sequencing analyses reveal a new regulator of virulence in *Agrobacterium fabrum* C58. Mol Plant Microbe Interact 28:580–589. doi:10.1094/MPMI-12-14-0380-FI. 26024442

[27] Lee K, Huang X, Yang C, Lee D, Ho V, Nobuta K, Fan JB, Wang K 2013 A genome-wide survey of highly expressed non-coding RNAs and biological validation of selected candidates in *Agrobacterium tumefaciens*. PLoS One 8:e70720. 23950988 10.1371/journal.pone.0070720 PMC3738593

[28] Möller P, Overlöper A, Förstner KU, Wen TN, Sharma CM, Lai EM, Narberhaus F 2014 Profound impact of Hfq on nutrient acquisition, metabolism and motility in the plant pathogen *Agrobacterium tumefaciens*. PLoS One 9:e110427. 25330313 10.1371/journal.pone.0110427 PMC4201532

[29] Wilms I, Overlöper A, Nowrousian M, Sharma CM, Narberhaus F 2012 Deep sequencing uncovers numerous small RNAs on all four replicons of the plant pathogen *Agrobacterium tumefaciens*. RNA Biol 9:446–457. doi:10.4161/rna.17212. 22336765 PMC3384568

[30] Chai Y, Winans SC 2005 A small antisense RNA downregulates expression of an essential replicase protein of an *Agrobacterium tumefaciens* Ti plasmid. Mol Microbiol 56:1574–1585. doi:10.1111/j.1365-2958.2005.04636.x. 15916607

[31] Overlöper A, Kraus A, Gurski R, Wright PR, Georg J, Hess WR, Narberhaus F 2014 Two separate modules of the conserved regulatory RNA AbcR1 address multiple target mRNAs in and outside of the translation initiation region. RNA Biol 11:624–640. doi:10.4161/rna.29145. 24921646 PMC4152367

[32] Wilms I, Voss B, Hess WR, Leichert LI, Narberhaus F 2011 Small RNA-mediated control of the *Agrobacterium tumefaciens* GABA binding protein. Mol Microbiol 80:492–506. doi:10.1111/j.1365-2958.2011.07589.x. 21320185

[33] Chevrot R, Rosen R, Haudecoeur E, Cirou A, Shelp BJ, Ron E, Faure D 2006 GABA controls the level of quorum-sensing signal in *Agrobacterium tumefaciens*. Proc Natl Acad Sci U S A 103:7460–7464. doi:10.1073/pnas.0600313103. 16645034 PMC1464361

[34] Wright PR, Richter AS, Papenfort K, Mann M, Vogel J, Hess WR, Backofen R, Georg J 2013 Comparative genomics boosts target prediction for bacterial small RNAs. Proc Natl Acad Sci U S A 110:E3487–E3496. doi:10.1073/pnas.1303248110. 23980183 PMC3773804

[35] Pain A, Ott A, Amine H, Rochat T, Bouloc P, Gautheret D 2015 An assessment of bacterial small RNA target prediction programs. RNA Biol 12:509–513. doi:10.1080/15476286.2015.1020269. 25760244 PMC4615726

[36] Wright PR, Georg J, Mann M, Sorescu DA, Richter AS, Lott S, Kleinkauf R, Hess WR, Backofen R 2014 CopraRNA and IntaRNA: predicting small RNA targets, networks and interaction domains. Nucleic Acids Res 42:W119–W123. doi:10.1093/nar/gku359. 24838564 PMC4086077

[37] Allison FE, Hoover SR, Burk D 1933 A respiration coenzyme. Science 78:217–218. doi:10.1126/science.78.2019.217. 17830277

[38] Streit WR, Entcheva P 2003 Biotin in microbes, the genes involved in its biosynthesis, its biochemical role and perspectives for biotechnological production. Appl Microbiol Biotechnol 61:21–31. doi:10.1007/s00253-002-1186-2. 12658511

[39] Feng Y, Zhang H, Cronan JE 2013 Profligate biotin synthesis in α-proteobacteria–a developing or degenerating regulatory system? Mol Microbiol 88:77–92. doi:10.1111/mmi.12170. 23387333 PMC3608792

[40] Vences-Guzmán MÁ, Guan Z, Bermúdez-Barrientos JR, Geiger O, Sohlenkamp C 2013 Agrobacteria lacking ornithine lipids induce more rapid tumour formation. Environ Microbiol 15:895–906. doi:10.1111/j.1462-2920.2012.02867.x. 22958119 PMC3772517

[41] Sheehan LM, Caswell CC 2018 An account of evolutionary specialization: the AbcR small RNAs in the *Rhizobiales*. Mol Microbiol 107:24–33. doi:10.1111/mmi.13869. 29076560 PMC5752129

[42] Dutcher HA, Raghavan R 2019 Origin, evolution, and loss of bacterial small RNAs, p 487–497. *In* Storz G, Papenfort K (ed), Regulating with RNA in bacteria and archaea. ASM Press, Washington, DC. doi:10.1128/microbiolspec.RWR-0004-2017.

[43] Updegrove TB, Shabalina SA, Storz G 2015 How do base-pairing small RNAs evolve? FEMS Microbiol Rev 39:379–391. doi:10.1093/femsre/fuv014. 25934120 PMC4542690

[44] Waters LS, Storz G 2009 Regulatory RNAs in bacteria. Cell 136:615–628. doi:10.1016/j.cell.2009.01.043. 19239884 PMC3132550

[45] Mann M, Wright PR, Backofen R 2017 IntaRNA 2.0: enhanced and customizable prediction of RNA–RNA interactions. Nucleic Acids Res 45:W435–W439. doi:10.1093/nar/gkx279. 28472523 PMC5570192

[46] Busch A, Richter AS, Backofen R 2008 IntaRNA: efficient prediction of bacterial sRNA targets incorporating target site accessibility and seed regions. Bioinformatics 24:2849–2856. doi:10.1093/bioinformatics/btn544. 18940824 PMC2639303

[47] Zuker M 2003 Mfold web server for nucleic acid folding and hybridization prediction. Nucleic Acids Res 31:3406–3415. doi:10.1093/nar/gkg595. 12824337 PMC169194

[48] Fröhlich KS, Vogel J 2009 Activation of gene expression by small RNA. Curr Opin Microbiol 12:674–682. doi:10.1016/j.mib.2009.09.009. 19880344

[49] Bossi L, Figueroa-Bossi N 2016 Competing endogenous RNAs: a target-centric view of small RNA regulation in bacteria. Nat Rev Microbiol 14:775–784. doi:10.1038/nrmicro.2016.129. 27640758

[50] Paulick A, Koerdt A, Lassak J, Huntley S, Wilms I, Narberhaus F, Thormann KM 2009 Two different stator systems drive a single polar flagellum in *Shewanella oneidensis* MR-1. Mol Microbiol 71:836–850. doi:10.1111/j.1365-2958.2008.06570.x. 19170881

[51] Torres-Quesada O, Millán V, Nisa-Martínez R, Bardou F, Crespi M, Toro N, Jiménez-Zurdo JI 2013 Independent activity of the homologous small regulatory RNAs AbcR1 and AbcR2 in the legume symbiont *Sinorhizobium meliloti*. PLoS One 8:e68147. doi:10.1371/journal.pone.0068147. 23869210 PMC3712013

[52] Hoffmann MC, Wagner E, Langklotz S, Pfänder Y, Hött S, Bandow JE, Masepohl B 2016 Proteome profiling of the *Rhodobacter capsulatus* molybdenum response reveals a role of IscN in nitrogen fixation by Fe-nitrogenase. J Bacteriol 198:633–643. doi:10.1128/JB.00750-15. PMC4751811 26644433

[53] Wilms I, Möller P, Stock AM, Gurski R, Lai EM, Narberhaus F 2012 Hfq influences multiple transport systems and virulence in the plant pathogen *Agrobacterium tumefaciens*. J Bacteriol 194:5209–5217. doi:10.1128/JB.00510-12. 22821981 PMC3457239

[54] Stepanek JJ, Schäkermann S, Wenzel M, Prochnow P, Bandow JE 2016 Purine biosynthesis is the bottleneck in trimethoprim-treated *Bacillus subtilis*. Proteomics Clin Appl 10:1036–1048. doi:10.1002/prca.201600039. 27329548

[55] Aiba H, Adhya S, de Crombrugghe B 1981 Evidence for two functional *gal* promoters in intact *Escherichia coli* cells. J Biol Chem 256:11905–11910. 6271763

[56] Waldminghaus T, Fippinger A, Alfsmann J, Narberhaus F 2005 RNA thermometers are common in α- and γ-proteobacteria. Biol Chem 386:1279–1286. doi:10.1515/BC.2005.145. 16336122

[57] Brantl S, Wagner EG 1994 Antisense RNA-mediated transcriptional attenuation occurs faster than stable antisense/target RNA pairing: an *in vitro* study of plasmid pIP501. EMBO J 13:3599–3607. doi:10.1002/j.1460-2075.1994.tb06667.x. 7520390 PMC395265

[58] Waldminghaus T, Heidrich N, Brantl S, Narberhaus F 2007 FourU: a novel type of RNA thermometer in *Salmonella*. Mol Microbiol 65:413–424. doi:10.1111/j.1365-2958.2007.05794.x. 17630972

