# Supplementary material for: A Small Regulatory RNA Controls Cell Wall Biosynthesis and Antibiotic Resistance
Source: mBio. 2018 Nov 13;9(6):e02100-18. doi: 10.1128/mBio.02100-18 (PMC6234868; doi:10.1128/mBio.02100-18)
Supplement: TABLE S1 [file mbo006184169st1.docx]

**Supplementary Table S1: Strains and plasmids used in this study.**

| **Strain or Plasmid** | **Relevant Characteristics** | **Reference or source** |
| --- | --- | --- |
| **Strains** |  |  |
| ***Escherichia coli*** |  |  |
| DH5α | Cloning host | (1) |
| ***Agrobacterium tumefaciens*** |  |  |
| C58 | wild-type | C. Baron, Montreal, Canada |
| C58 ∆PmaR | Derivate of the wild-type with deletion of the PmaR gene | This study |
| **Plasmids** |  |  |
| pUC18 | Amp^r^; cloning vector | (2) |
| pK19*mobsacB* | Km^r^; suicide vector | (3) |
| pSRK | Km^r^; complementation vector | (4) |
| pK19_up/do_PmaR (pBO2034) | Km^r^; pK19*mobsacB* derivate carrying the up- and downstream regions of PmaR | This study |
| pSRK_PmaR (pBO4512) | Km^r^; pSRK derivate carrying PmaR | This study |
| pSRK_Mut (pBO4524) | pBO4512 carrying site-directed mutagenesis of PmaR (58-61 CCCA-TTTT) | This study |
| *murB*_*lacZ* (pBO4538) | Amp^r^; Gm^r^_;_ pUC19 derivate carrying transcriptional fusion of *murB* (*atu2092*) to *lacZ*; OriT | This study |
| *cheD*_*lacZ* (pBO4541) | Amp^r^; Gm^r^_;_ pUC19 derivate carrying transcriptional fusion of *cheD* (*atu2618*) to lacZ; OriT | This study |
| *ampC*_*lacZ* (pBO4542) | Amp^r^; Gm^r^_;_ pUC19 derivate carrying transcriptional fusion of *ampC* (*atu3077*) to lacZ; OriT | This study |
| *atu3504_lacZ* (pBO4578) | Amp^r^; Gm^r^_;_ pUC19 derivate carrying transcriptional fusion of *atu3504* to lacZ; OriT | This study |
| *murB*_3×FLAG (pBO4559) | Amp^r^; Km^r^_;_ pUC19 derivate carrying translational fusion of *murB* (*atu2092*) with 3×FLAG; OriT | This study |
| *cheD*_3×FLAG (pBO4557) | Amp^r^; Km^r^_;_ pUC19 derivate carrying translational fusion of *cheD* (*atu2618*) with 3×FLAG; OriT | This study |
| *ampC*_3×FLAG (pBO4558) | Amp^r^; Km^r^_;_ pUC19 derivate carrying translational fusion of *ampC* (*atu3077*) with 3×FLAG; OriT | This study |
| *atu3504*_3×FLAG (pBO4567) | Amp^r^; Km^r^_;_ pUC19 derivate carrying translational fusion of *atu3504* with 3×FLAG; OriT | This study |
| runoff_PmaR (pBO4560) | Amp^r^; pUC19 derivate carrying PmaR with additional T7-promotor | This study |
| runoff_PmaR Mut (pBO4569) | pBO4560 carrying site-directed mutagenesis of PmaR (58-61 CCCA-TTTT) | This study |
| runoff_*ampC* (pBO4570) | Amp^r^; pUC19 derivate carrying *ampC* (+175/+325 AUG) with additional T7-promotor | This study |
| runoff_*murB* (pBO4572) | Amp^r^; pUC19 derivate carrying *murB* (-100/+67 AUG) with additional T7-promotor | This study |

1. Hanahan D. 1983. Studies on transformation of *Escherichia coli* with plasmids. J Mol Biol 166:557-580.

2. Norrander J, Kempe T, Messing J. 1983. Construction of improved M13 vectors using oligodeoxynucleotide-directed mutagenesis. Gene 26:101-106.

3. Schäfer A, Tauch A, Jäger W, Kalinowski J, Thierbach G, Pühler A. 1994. Small mobilizable multi-purpose cloning vectors derived from the *Escherichia coli* plasmids pK18 and pK19: selection of defined deletions in the chromosome of *Corynebacterium glutamicum*. Gene 145:69-73.

4. Torres-Quesada O, Millán V, Nisa-Martínez R, Bardou F, Crespi M, Toro N, Jiménez-Zurdo JI. 2013. Independent Activity of the Homologous Small Regulatory RNAs AbcR1 and AbcR2 in the Legume Symbiont *Sinorhizobium meliloti*. PLoS ONE 8:e681.
