# Supplementary material for: A Small Regulatory RNA Controls Cell Wall Biosynthesis and Antibiotic Resistance
Source: mBio. 2018 Nov 13;9(6):e02100-18. doi: 10.1128/mBio.02100-18 (PMC6234868; doi:10.1128/mBio.02100-18)
Supplement: TABLE S2 [file mbo006184169st2.docx]

**Supplementary Table S2: Oligonucleotides used in this study.**

Restriction sites are underlined and T7 promoter sequences for generation of *in vitro* transcripts are written in bold letters. Exchanged nucleotides used for site-directed mutagenesis are marked in grey.

| **Oligonucleotide** | **Purpose** | **Sequence (5‘ to 3‘)** |
| --- | --- | --- |
| pSRK_PmaR_fw | cloning of PmaR in pSRK | AAAAGGATCCTTGTCAATTGATGCCTAGGCAC |
| pSRK_PmaR_rv |  | AAAAGAGCTCTGGAAGATCCGATCCGGG |
| PmaR_QC_fw | PmaR site-directed mutagenesis (58-61 CCCA-TTTT) | GCATTGACCCTTTACCTTTTGCCCCCAATGCCCGGATCGG |
| PmaR_QC_rv |  | CCGATCCGGGCATTGGGGGCAAAAGGTAAAGGGTCAATGC |
| RNAprobe_PmaR_fw | Northern analysis; RNA-probe for PmaR | TGTCAATTGATGCCTAGG |
| RNAprobe_PmaR_rv |  | **GAAATTAATACGACTCACTATAGGG**TGGAAGATCCGATCCGG |
| RNAprobe_*murB*_fw | Northern analysis; RNA-probe for *murB* (*atu2092*) | GGACGAAAACCGCATCAAGG |
| RNAprobe_*murB*_rv |  | **GAAATTAATACGACTCACTATAGGG**CCTTGTCTTCCGGATAACCC |
| RNAprobe_*murI*_fw | Northern analysis; RNA-probe for *murI* (*atu1867*) | TGAAGACGCGCATTCTCTCG |
| RNAprobe_*murI*_rv |  | **GAAATTAATACGACTCACTATAGGG**CTGATCCAGCTTTCGGCCAT |
| RNAprobe_*cheD*_fw | Northern analysis; RNA-probe for *cheD* (*atu2618*) | TCTCAGGCCACCATCGAGTT |
| RNAprobe_*cheD*_rv |  | **GAAATTAATACGACTCACTATAGGG**CTGCAAAGCATCGGCAAGCA |
| RNAprobe_*3504*_fw | Northern analysis; RNA-probe for *atu3504* | TAACCAGGTGACGGATGTCG |
| RNAprobe_*3504*_rv |  | **GAAATTAATACGACTCACTATAGGG**CACTGGCGTGTTGTCGAAGA |
| RNAprobe_*bioA*_fw | Northern analysis; RNA-probe for *bioA* (*atu4000*) | TTCCGATAGCGGTTCAACGG |
| RNAprobe_*bioA*_rv |  | **GAAATTAATACGACTCACTATAGGG**GCTAGAACATTGGCGGCGTA |
| RNAprobe_*ampC*_fw | Northern analysis; RNA-probe for *ampC* (*atu3007*) | ATGTCGCGCTGATGCATCTC |
| RNAprobe_*ampC*_rv |  | **GAAATTAATACGACTCACTATAGGG**GAGCCGGTTCACCTGTTCTT |
| *murB*_SalI_fw | cloning of *murB*_*lacZ* and *murB*_3×FLAG | TTTTGTCGACAGCTCGGAAGCGCTGGTTT |
| *murB*_BamHI_rv | cloning of *murB*_*lacZ* | TTTTGGATCCTTACGCCCCGAGGAACG |
| *murB*_3×FLAG_rv | cloning of *murB*_3×FLAG | GGGCGCCCCGAGGAACGGCT |
| *cheD*_SalI_fw | cloning of *cheD*_*lacZ* and *cheD*_3×FLAG | TTTTGTCGACCTAATATTAAAAGCCAACGAACAGT |
| *cheD*_XbaI_rv | cloning of *cheD*_*lacZ* | TTTTTCTAGATTAGAATTCCGCCCATTCGG |
| *cheD*_3×FLAG_rv | cloning of *cheD*_3×FLAG | GGGGAATTCCGCCCATTCGGC |
| *ampC*_SalI_fw | cloning of *ampC*_*lacZ* and *ampC*_3×FLAG | TTTTGTCGACGGGCGGCTAAACGTCTTTTC |
| *ampC*_BamHI_rv | cloning of *ampC*_*lacZ* | TTTTGGATCCTTATTGCGCCTTTTCGAGCG |
| *ampC*_3×FLAG_rv | cloning of *ampC*_3×FLAG | GGGTTGCGCCTTTTCGAGCGC |
| *atu3504*_SalI_fw | cloning of *atu3504*_*lacZ* and *atu3504*_3×FLAG | TTTTGTCGACCAAAGCACGACTATTTCTCGTC |
| *atu3504*_BamHI_rv | cloning of *atu3504*_*lacZ* | TTTTGGATCCTTAACGGCTGCCGTAAAGCG |
| *atu3504*_FLAG_rv | cloning of *atu3504*_3×FLAG | GGGACGGCTGCCGTAAAGCGTAT |
| Runoff_PmaR_fw | plasmid generation for PmaR runoff transcription | AAAAG**GAAATTAATACGACTCACTATAGGG**TTGTCAATTGATGCCTAGGCA |
| Runoff_PmaR_rv |  | TTTTGATATCTGGAAGATCCGATCCGGG |
| Runoff_*murB*_fw | plasmid generation for *murB* runoff transcription | AAAAG**GAAATTAATACGACTCACTATAGGG**CGAATCCGGGTGATTTTGTG |
| Runoff_*murB*_rv |  | TTTTGATATCGTCCCCGCAATTCATTTACC |
| Runoff_*ampC*_fw | plasmid generation for *ampC* runoff transcription | AAAAG**GAAATTAATACGACTCACTATAGGG**GCTGGGGATGGCGGAGAA |
| Runoff_*ampC*_rv |  | TTTTGATATCGCCTTCAGCCTCGCATCATC |
